# Orf with photoaggravated eruption

**DOI:** 10.1016/j.jdcr.2021.08.010

**Published:** 2021-08-14

**Authors:** Christoffer Aam Ingvaldsen, Erling Undersrud, Jon Gottfred Andersen

**Affiliations:** a Department of Dermatology, Rikshospitalet, Oslo University Hospital, Oslo, Norway; b Department of Pathology, Stavanger University Hospital, Stavanger, Norway; c Department of Dermatology, Stavanger University Hospital, Stavanger, Norway

**Keywords:** orf, orf virus, parapoxvirus, echtyma contagiosum, contagious pustular dermatitis, scabby mouth, viral mimicry, hypersensitivity, photoaccentuation, photoaggravation, photodistribution, photosensitivity, EM, erythema multiforme, PLE, polymorphic light eruption

## Introduction

Orf could be seen as an occupational dermatosis among sheep farmers and shepherds. It is caused by a zoonotic DNA parapoxvirus transmitted to humans from sheep and goats.^1^ It resembles “Milker's nodule” which is caused by another parapoxvirus infecting the teats of cows.^2^

Orf virus transmission occurs through inoculation of broken or abraded skin- either by direct contact with affected animals or indirectly with contaminated fomites.^3^ The virus is resistant to drying and freezing and may remain viable on inanimate objects for months to years.^4^ Repeated outbreaks are normal, and larger case series from Norway and New Zealand have been published.^2^^,^^3^ In the United Kingdom, approximately 30% of shepherds reported previous infection with orf.^5^

A digital nodule presenting 3 to 7 days after inoculation is characteristic of orf. The lesion ulcerates, forms a dry crust, and resolves within 4 to 8 weeks.^1^^,^^2^ Its benign and self-limiting nature is known among most people handling sheep.^3^^,^^5^

There is a growing body of literature reporting systemic hypersensitivity reactions caused by orf virus. Such hypersensitivity reactions resemble cell-mediated immune reactions seen in herpes simplex infections. Erythema multiforme (EM) is the most frequent and observed in 7% to 18%.^1^ Moreover, widespread papulovesicular eruptions and immunobullous dermatoses have been reported.^1^^,^^2^^,^^6^ Dermatologists should be aware of orf and the wide array of hypersensitivity reactions it can cause. Here, we describe 2 cases of orf with photoaggravated eruptions.

## Case report

In spring, 2 unrelated sheep farmers from the southwest coast of Norway presented with similar skin changes. Patient 1 was a 32-year-old, healthy woman with nickel allergy. Patient 2 was a 42-year-old woman with erenumab-treated migraine and sulfonamide (exanthema) and birch pollen (rhinitis) allergies. None of the patients had otherwise any underlying dermatoses. Both patients had observed oral and perioral lesions (“scabby mouth”) among their sheep prior to presentation.

**Fig 1:**
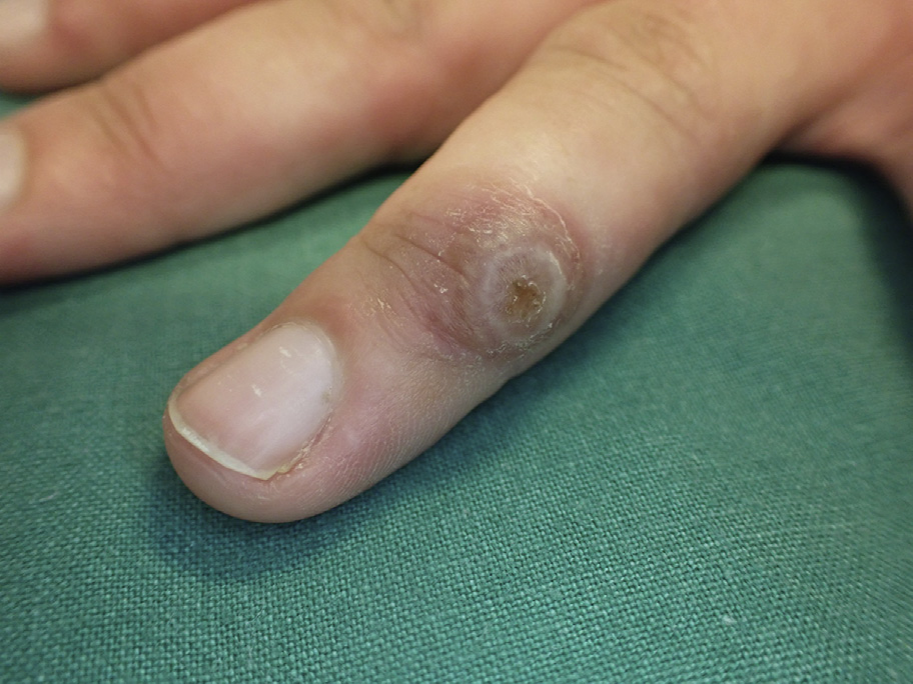
Primary lesion (“echtyma contagiosum”) on the index finger of patient 1.

**Fig 2:**
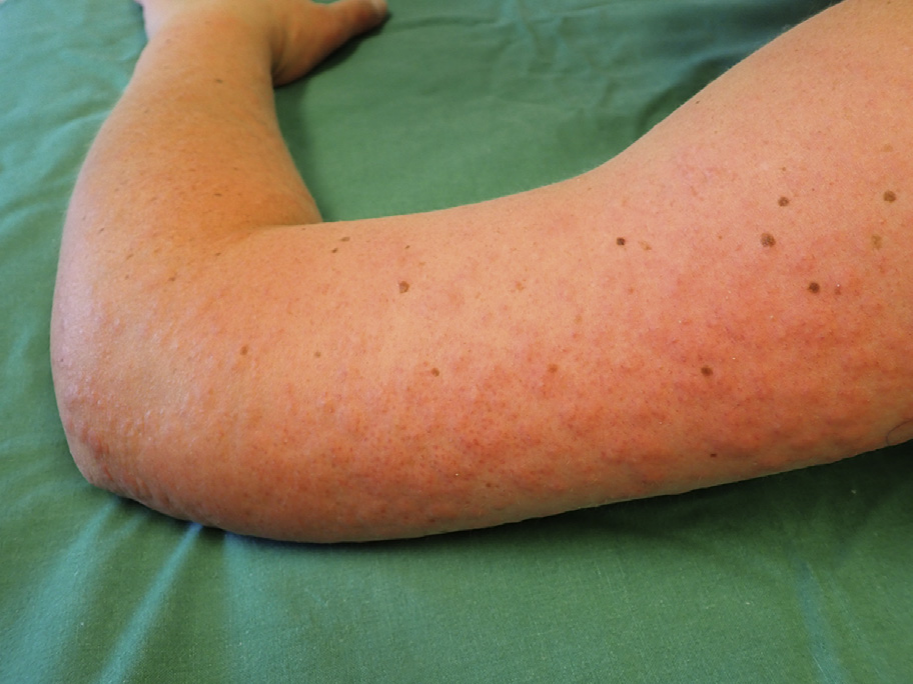
Erythematous, papulovesicular, and urticarial eruption on dorsolateral aspect of the upper extremities of patient 1. Patient 2 had similarly distributed eruption with more evident vesicles.

Patient 1 presented with a crusted nodule (primary lesion) on the dorsal aspect of her left ring finger (Fig 1) and a symmetric, polymorphic, and photoaggravated eruption. The dorsolateral aspects of the upper extremities were most prominently affected (Fig 2); however, similar lesions were also seen on sun-exposed skin on her back and distal extremities. In addition, multiple annular and urticarial skin changes were observed in the palms of the patient. These bore resemblance to targetoid lesions as might be seen in EM; however, a maximum of 2 concentric color zones were seen. The crusted nodule had presented 8 to 10 days prior to the eruption.

**Fig 3:**
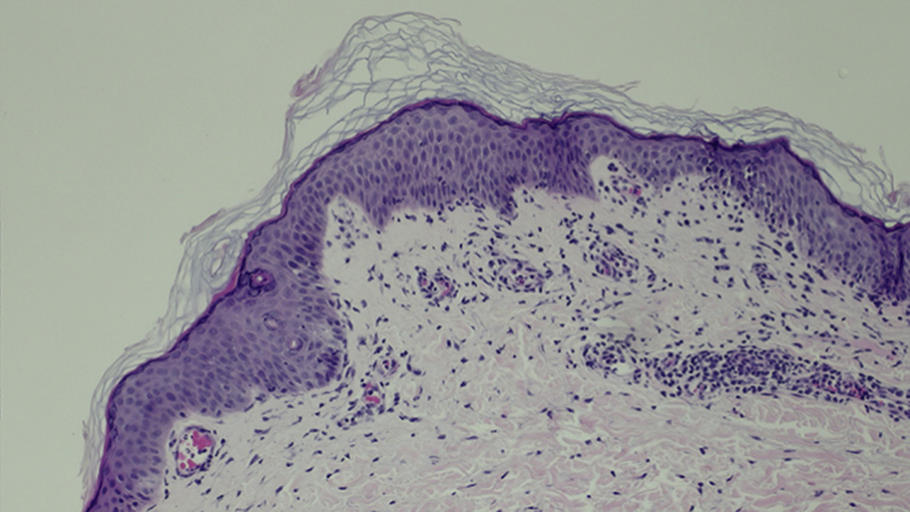
Skin biopsy from shoulder region showing subtle spongiosis with lymphoid exocytosis, focal papillary dermal edema, and urticaria-like changes in the superficial dermis. (Hematoxylin-eosin stain; original magnification: ×10.)

Two punch biopsies from the shoulder area were performed, both demonstrating similar features of the following (Fig 3): slight spongiosis, focal vacuolization of the dermoepidermal junction without keratinocyte necrosis, and a superficial perivascular lymphocytic infiltrate with papillary edema containing neutrophils and eosinophils, resembling urticaria. The histologic picture did not have features of EM.

**Fig 4:**
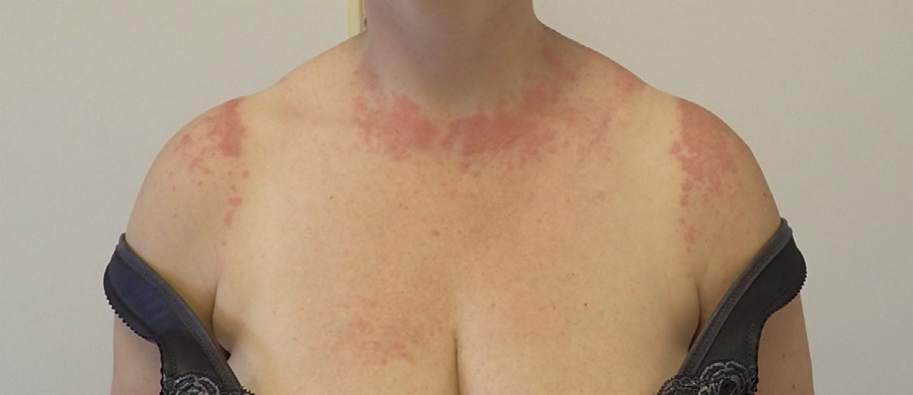
Photoaggravated eruption in patient 2.

Patient 2 presented with almost identical polymorphic skin changes and distribution. The primary lesion, localized to the left index finger, had presented approximately 14 to 18 days prior to the eruption. As with patient 1, palmar, EM-like lesions were observed. The patient had worn a tank top, which had resulted in sharply demarcated borders between affected and unaffected skin (Fig 4).

Both patients reported only mild tenderness from the primary lesion; however, the eruptions were highly pruritic. No mucosal skin changes were seen. In both cases, the face was unaffected. No systemic symptoms other than pruritus were recorded.

The eruptions were successfully managed with 60-mg prednisolone once daily for 5 days, combined with 5- to 10-mg desloratadine twice daily for 2 weeks and topical clobetasol proprionate tapered over 3 weeks. The primary lesions did not fully resolve before an additional 4 to 6 weeks.

## Discussion

The dermatotropic orf virus replicates in the cytoplasm of cutaneous cells and produces an arsenal of virulence factors with immunomodulatory mechanisms.^4^ Viral DNA fragments are typically released from infected cells.^4^ As such, both viral virulence factors and DNA fragments are presented in the skin. These have molecular earmarks of mammalian proteins.^1^ A homolog of mammalian vascular endothelial growth factor is, for instance, an important virulence factor.^1^ This viral mimicry makes it difficult for the host's immune system to distinguish between host and viral proteins. The result is autoreactive T cells causing hypersensitivity reactions outside the primary lesion.^1^

Several hypersensitivity reactions caused by orf virus have been reported.^1^^,^^2^^,^^6^ Although EM is the most common, the clinical and histopathologic features outlined in our patients do not point in a direction of photoaggravated EM. Orf-induced polymorphic light eruption (PLE), especially the EM-like subgroup, is another plausibility.^7^ PLE, however, is unlikely due to the distribution of the lesions (palmar involvement, faces unaffected), as well as the absence of the superficial and deep “coat-sleeve” infiltrate on biopsy that typically characterizes PLE.

On the basis of an extensive literature review, we believe that these eruptions could represent viral dissemination, followed by a photoaggravated hypersensitivity reaction. This is reported in other viral diseases (eg, herpes simplex, varicella, and echovirus 9).^8^^,^^9^

The potential mechanisms of photoaggravation in viral disease have been discussed.^8^, ^9^, ^10^ Theoretically, ultraviolet radiation could trigger and/or aggravate eruptions in viral disease through the following mechanisms: (1) cutaneous inflammation with increased capillary permeability and deposition of viral particles, (2) immunosuppression, (3) increased viral proliferation caused by thermal cell membrane damage, (4) virus dissemination by infected melanocytes distributing melanin, and (5) binding of infected lymphocytes to keratinocytes as a result of enhanced expression of intercellular adhesion molecule 1.^9^^,^^10^

The 2 reported cases demonstrate that viral disease may mimic a photodermatosis. Unfamiliarity with this phenomenon may lead to misdiagnosis, such as PLE, in such patients.

## Conflicts of interest

None disclosed.
